# Sertoli leydig cell tumor of the ovary in a woman with cushing syndrome: A case report

**DOI:** 10.1016/j.gore.2023.101277

**Published:** 2023-09-27

**Authors:** Tuba Ofli, Gurkan Kiran, Atilla Kunt

**Affiliations:** a Department of Obstetrics and Gynecology, Basaksehir Cam and Sakura City Hospital, Istanbul, Turkey; b Department of Obstetrics and Gynecology, Bezmialem University, Faculty of Medicine, Istanbul, Turkey; c Department of Obstetrics and Gynecology, Medeniyet University, Faculty of Medicine, Istanbul, Turkey

**Keywords:** Sertoli leydig cell tumor, Cushing's syndrome, Androgenism, Paraplegia

## Abstract

Sertoli-Leydig cell tumor (SLCT) is a rare tumor of the ovary.Cushing's syndrome (CS), on the other hand, is a clinical picture formed by the long-term high levels of glucocorticoids in the blood for any reason and the resulting symptoms. Exceptionally in some of cases, a tumor far from the adrenal region synthesizes adrenocortical hormones. Among such ectopic neoplasms, CS Tumors of the ovary that secrete cortisol as a cause of the disease is an exceptional case. In other words, in this case, we argue that the tumor in the ovary causes Cushing's syndrome by secreting cortisol and ACTH-like peptides. There are 5 cases reported in the literature. In this case report we present a case in which SCLT of the ovary was detected by histopathological examination in a patient who underwent laparoscopic surgery due to Cushing's syndrome and bilateral adnexal mass.

## Highlights

• In the subgroup of ovarian sex cord stromal tumor symptoms related to hormonal disorders (androgenic, estrogenic) may be seen.
• Among ectopic neoplasms, ovarian tumors that secrete cortisol, causing Cushing's syndrome, are exceptional.
• The prognosis may be poor as mortality may result from metabolic disorders such as severe hypokalemia and ketoacidosis.
• The risk of metastatic disease and thromboembolism are important factors in the follow-up process.

## 1 Introduction

Sertoli-Leydig tumors are defined as tumors consisting of a primal stroma and heterologous elements with varying rates of Sertoli and Leydig cells by WHO. (Tavassoli et al., 2003) SLCT are rare tumors that make up less than 0.2% of all ovarian cancers. SLCTs are approximately 7% of all ovarian neoplasms and are classified under sex cord-stromal tumors (SCST)**.** (Colombo et al., 2007) Most common symptoms are abdominal pain, swelling, and hormonal (such as androgenic, estrogenic) changes. High levels of exposure to androgens can cause oligomenorrhea amenorrhea and hirsutism. Moreover, virilization findings such as male-type hair loss, clitoromegaly and deepening of the voice can also be seen.

CS is defined as having elevated blood glucocorticoid levels for an extended amount of time and the signs and symptoms caused by the elevated glucocorticoid levels. If the elevation of the glucocorticoids is caused by the increase of ACTH secreted by the pituitary gland then it is called Cushing disease, if the elevation is caused by other sources it is called “Cushing Syndrome” Bilateral adrenocortical hyperplasia can occur due to high ACTH level can be seen in 60% to 70% of all the Cushing Syndrome cases (Bhansali et al., 2009) 30–40% of the cases Cushing syndrome can be caused by benign or malignant tumors found in adrenal glands; independent of ACTH levels. (Bovio et al., 2009, Pollack and Brett, 2009, Louiset e Gobet et al., 2010) However, in some rarely seen cases, a tumor distant from the adrenal region can perfectly mimic adrenal secretion by synthesizing adrenocortical hormones. Cortisol-secreting ovarian tumors causing CS are exceptions among ectopic neoplasms. (Marieb et al., 1983, Young and Scully, 1987, Donovan et al., 1993, Elhadd et al., 1996).

In this case report we present a case in which SCLT of the ovary was detected by histopathological examination in a patient who underwent laparoscopic surgery due to Cushing's syndrome and bilateral adnexal mass.

## 2 Case report

The 21-year-old patient, who was administered 8 mg betamethasone for tocolysis due to the threat of preterm labor during pregnancy, performed preterm normal delivery in the thirty-second gestational week. She applied to an outside clinic with complaints of inability to walk, swelling of the face, edema and rash on the body 2 months postpartum, where a pelvic USG was performed. Pelvic MRI was performed with suspicion of a mass in the ovary; nodular lesions filling both adnexal lodges were 72x60 mm on the left and 64x55mm on the right (T1a hypointense T2a mild hyperintense showing contrast enhancement after intravenous contrast application). Complaints of the patient when he applied to our hospital from the emergency department; There were swelling, weakness, acne, increased hair growth, purple spots on the abdomen, arms and legs, decreased speech, inability to walk, increased thirst, and frequent urination.

Clinical findings in the internal medicine consultation requested from the emergency department; edema of the face and neck region, papulopustular lesions on the chest, virilization around the navel and aerola, distended abdomen, purple striae on the chest and upper extremity.

Laboratoryresults;Cortisol:54.8ug/dL. ACTH:209 pg/mL .TSH:0.7 uIU/mL PTH(Parathormone):27.9 pg/mL Potassium: 1.78 mmol/L.

A preliminary diagnosis of 'Cushing's syndrome' was reached with the examinations and clinical findings.. Dexamethasone suppression test was applied to confirm. Results are presented in Table 1. Tumor markers were found slightly elevated (CA 125:57.47 U/mL, CA 19–9:22.08U/mL). Cranial MR showed T1a and T2a isointense microadenoma? 4 mm in diameter.

**Table 1:** Dexamethasone suppression test results of the patient.

Hormone	Base	Deksamethasone			Reference Values
		1 mg	2 mg	8 mg	
Cortisol(ng/mL)	54.8	51	35.4	32.3	50-250
ACTH (pg/mL)	209				20–46

Hypokalemia on admission was detected (1.78) and appropriately replaced. To diagnose the cause of the patient’s paraplegia Lomber MR was performed with the recommendation of Orthopedics and Traumatology. MR revealed compression fractures on T8-9 and L2-4. Due to the normal size of the adrenal glands, absence of skin hyperpigmentation, and higher than expected plasma ACTH levels, KS originating from an ectopic focus was considered to be the cause of the clinical picturenormal-sized. Brain CT was performed as a result of the neurosurgery consultation requested due to the microadenoma detected in the pituitary; there was no pathology observed. In other imaging, both adrenal glands were normal size and did not contain any mass or tumors, so pelvic tumor-induced ectopic glucocorticoid production was suspected. To evaluate ectopic Cushing syndrome and to identify the source PET-CT imaging was done. Due to PET-CT pointing to primary ovarian cancer, the patient was consulted to Gynecologic Oncology. Surgical excision of the mass was decided and the patient was then transferred to our clinic.

**Fig. 1:**
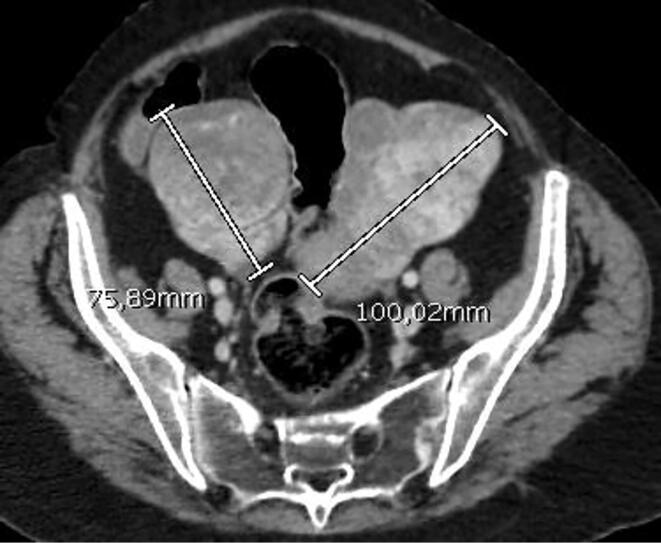
In the axial image of the whole abdominal CT with IV contrast, heterogeneous solid lesions enhanced by contrast with lobulated contours are seen in both adnexal regions.

Preop gastroscopy and colonoscopy were performed because the patient had bilateral ovarian masses. However, the results were not significant and did not point to another primary cancer or metastasis. In the lower abdomen CT, a nodular tumoral mass was observed to fill both adnexal lobes, with a lobulated configuration of 95x85 mm on the right with the largest of the solid nodules reaching 6 cm in diameter on the right tumoral mass, and a lobulated mass of 83x72 mm on the left adnexa (mass evaluated in favor of serous ovarian cancer**) (**Fig. 1**)** Pathologic lymph nodes were not detected in the pelvic or paraaortic region. Bloodwork showed the tumor markers as CEA:8U/mL, CA 125: 64U/mL, CA15-3:25U/mL.

The surgery was planned as Diagnostic Laparoscopy, and if required followed by Total Abdominal Hysterectomy + Bilateral Salpingo Oophorectomy + Frozen. After the required preoperative preparations, the patient was operated on;

The frozen response received at the surgery was 'Granulosa Cell Tumor'. Therefore, hysterectomy + bilateral salpingo-oophorectomy was performed.

Both adnexa were sent for frozen study. In the macroscopic examination of the right and left adnexa; A lobulated contoured, encapsulated, yellow-pink colored mass with soft elastic consistency was observed. The cross-sectional surface of the masses was observed as yellow-pink colored and occasionally dirty yellow necrotic nodules. In addition, in the sections of the hysterectomy sent later, the endometrium was 0.1 cm; Myometrium is 1.5 cm thick and no feature is observed. In the microscopic examination of the samples taken from the masses; In the fibrohyalinized stroma, cells with lobular pattern, solid islands and vesicular chromatin forming an open glandular structure, irregular membrane nuclei, large eosinophil cytoplasm, and some bizarre-looking atypia were observed. Calcification areas were observed in the middle of the gland structures and in the stroma in places. In the tumor, there were 3–4 mitosis, tumor necrosis and widespread lymphatic invasion in 10 high magnification fields (3–4/hpf). The residual ovarian cortex around the tumor and the adjacent 'Adrenal Rest' area were noted.

**Fig. 2:**
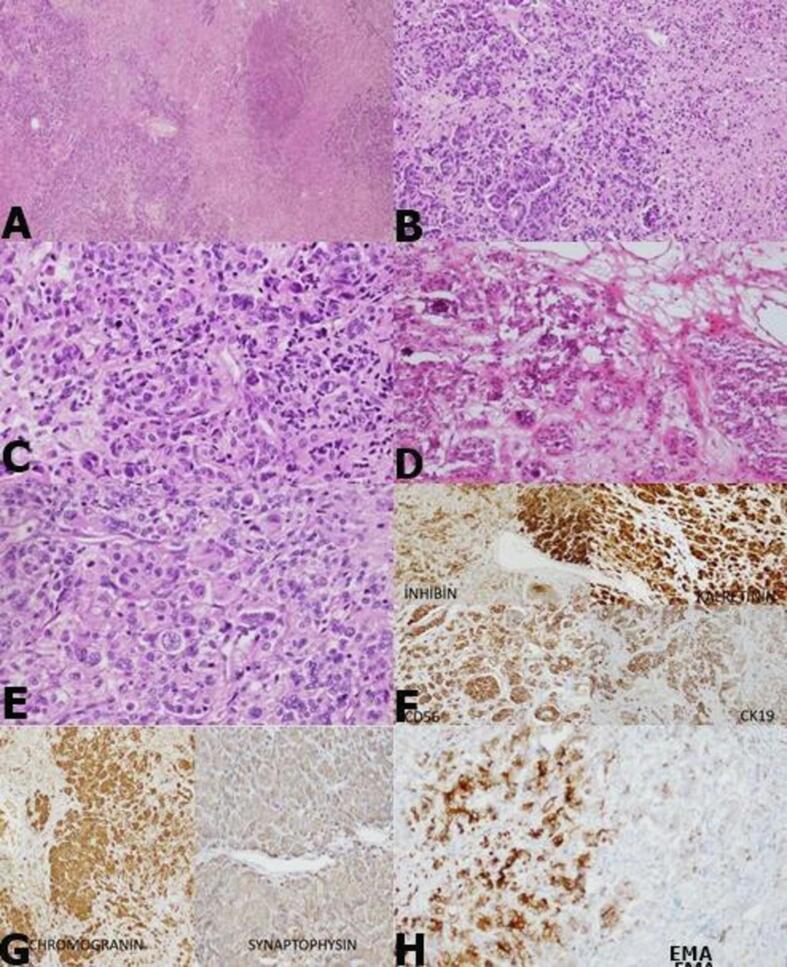
Lobular and necrotic nodular formation of the masses (A: H&Ex40), cells with degenerative atypia forming solid islands and open lumen gland structure (B: H&Ex100), vesicular chromatin forming solid islands in hyalinized stroma (C: H&Ex200), cytoplasm with irregular membrane nuclei, large eosinophils atypical cells (D: H&Ex100, E:H&Ex200) Immunohistochemical stainings) (F: inhibin, calretinin, CD56, CK19. G: Chromogranin, synaptophysin H: EMA positivity is observed).

In the immunohistochemical studies applied to the case, atypical cells showed positivity with Panck, Cytokeratin19, CEA (focal), Inhibin, Calretinin, Chromogranin, Synaptophysin, CD56, EMA, PIT-1, ACTH, MUC5AC (focal); Cytokeratin7, Cytokeratin20, OCT-4, CD30, PLAP, TAG-72, CA125, WT-1, Melan-A, CD117, were negative with PR. Ki67 proliferation index was detected at the rate of 70–80% in the densest area. **(**Fig. 2).

The patient was transferred to the Intensive Care Unit for post-op follow-up. The patient, whose follow-up and treatment were carried out; upon the improvement of the patient was transferred to our clinic 2 days later. The cushingoid appearance of the patient improved rapidly, and the edema on his face and body, along with the rashes and the striae regressed. Laboratory values improved to normal values. Her electrolytes have reached normal levels. (Table 2) The patient, whose post-op care was completed, was transferred to the Endocrine service on post-op day five.

**Table 2:** Pre-operative and post-operative laboratory results of the patient.

Routine analysis	PRE-OP	POST-OP	Reference values
Glucose (mg/dL)	220	101	70-105
BUN (mg/dL)	13.08	4.6	7-18
Creatinine (mg/dL)	0.72	0.48	0.57-1.1
Cortisol (µg/dL)	34.09	8.3	3.7-19.4
Sodium (mmol/L)	142	140	135-145
Potassium (mmol/L)	2.1	3.85	3.5-5.1
Hemoglobin (g/dL)	13.2	10.2	13-16
Hematocrite	39.5	30	35-48
ACTH (pg/mL)	89	16	< 46
ALT (U/L)	83	29	0–55
AST(U/L)	24	19	5–34

In the laboratory tests performed in the endocrine service, cortisol checked at night was 8 µg / dL, and basal cortisol level was 13 ug / dL. As the cortisol level decreased and improvement was observed in the patient's clinical table, the patient was discharged by the Department of Endocrinology after planning the outpatient follow-up care of the patient.

## 3 Dıscussıon

SLCTs are typically unilateral tumors. Only 1.5% of them occur bilaterally, as seen in our case. Most tumors are limited to the ovaries and 97.5% are stage I at the time of diagnosis. (Young and Scully, 1987).

SLCTs are characterized by the presence of testicular structures (Sertoli and Leydig cells) capable of producing androgens. SCLTs can simply cause virilization, or it may progress to aggressive metabolic activity that is enough to cause Cushing syndrome.

The prognosis is linked to the degree of cell differentiation and the presence of heterologous elements. Histologically, SLCTs are divided into good, moderate, and poorly differentiated tumor subgroups based on cytological atypicality, mitotic activity, presence or absence of sarcomatous growth pattern. Heterologous elements (chondroid, leiomiogenic, rhabdomyogenic, gastrointestinal type, or carcinoid differentiation) usually accompany moderately and poorly differentiated subtypes, however are also rarely seen in well-differentiated tumors. (Stacher et al., 2010).

Diagnostic accuracy is postoperative histological and, despite strong clinical assumptions, there are no specific ultrasound findings. SLCT's ultrasound images can vary. On ultrasound, Sertoli-Leydig cell tumors appear as heterogeneous masses of vascularized tissue containing solid areas; in SLCT forms consist of pure Sertoli cells, they are readily localized in relation to dense fluid regions; their sizes are variable, reaching around twenty centimeters**.** (Demidov et al., 2008) Demidov analyzed 22 patients (15 SLCT, 2 Sertoli cell tumors, 5 Leydig cell tumors) and showed that 15 of the 22 patients had solid mass images, 6 had a multicystic, lobulated image with solid areas, and 1 had a multicystic-multilocular image. (Demidov et al., 2008).

Cushing's syndrome is rarely associated with ovarian neoplasms. Although the incidence of Cushing syndrome due to ovarian neoplasms is not known, in case reports, they defined the mechanisms of ACTH secretion as the ectopic production of ACTH-like peptides and the release of corticotropin release factor and cortisol. (Donovan et al., 1993)In these cases, the prognosis may be poor as mortality may result from metabolic disorders such as severe hypokalemia and ketoacidosis. However, the most threatening causes are metastatic disease and thromboembolism (Marieb et al., 1983). Six cases reported in the literature resulted in death (Van der Pas et al., 2013, Marieb et al., 1983, Young and Scully, 1987, Donovan et al., 1993, Elhadd et al., 1996)**.** The mentioned cases are presented in Table 3. No cases with brain metatastases have been found in the literature (Colombo et al., 2007)**.**

**Table 3:** Case of malign over ca releasing cortisole published in the literature, includıng current case.

Case	Age	Clinical table	Laboratory results	Acth	Imaging	Operation	Pathology	Prognosis
(Marieb et al., 1983)(9)	35	- Cushing syndrome - Severe Virilization - Peripheral edema - Amenorrhea - Lower Abdominal mass - BP = 150/80 mmHg	-Ürinary and plasma cortisole- Urinary 17-hydroxysteroids and 17-ketosteroids- higher than plasma levels Low FSH and LH	Low = 28 pg / mL (20–100)	Bilateral ovarian mass. No adrenal mass, adrenals are of normal size	Bilateral ovarian mass Omentum cul-de-sac and mesentery implants Normal adrenals	Right ovarian Carcinoma = 15 cm Left one = 8 cm Widespread metastasis Classification: Ovarian Tumour of malignant steroid cells	Normalization of steroids followed by recurrent chemotherapy failure, exitus after 17 months
Young *et al*(1987) (10)	48	- Cushing syndrome - Light virilization - BP: 180/110 mmHg - Diabetes mellitus	Increased cortisole	–	Right Adnexial Mass	Yellow brown colored ovary. Yellow brown nodules on visceral and parietal peritoneum. Omentum and diaphragma normal	steroid cell (lipid cells) type abdominal metastesesı	Normalization of steroids and blood pressure. Recurrence on month 3 chemotherapy failure, exitus on month 10
Donovan *vd*.(1993) (11)	66	Abdominal-pelvic mass - Lower extremity edema - Diabetes mellitus Cushing syndrome + Post operational Hipertension	Hipokalemia increased - CA125 - Plasma and urine cortisol - DHEA - S - Testosteron - Androstenedione	Normal	–	The small intestine has several attached loops 8 cm multiloculated right ovarian tumor	Ovarian Tumors of malignant steroid cells Metastases: colon and intestines, omentum and liver	Ketoconazole and classic chemotherapy failure, exitus in 4 months
Young and Scully vd(1987)(10)	52	- Cushing syndrome - Hirsutism - High Blood pressure for 17 years(170/100) - Diabetes mellitus	Increased Ürinary and plasma cortisol- Urinary 17-hydroxysteroids and 17-ketosteroids-	Normal	Noırmal venographic imaging of adrenals	Right ovarian tumor,Widespread metastases to omentum	Malignant ovarian tumor with an intact capsule 135 gr (9 × 7 × 5 cm) Malignant looking omental lesions	Exitus in 6 months
Elhadd *vd*.(1996) (12)	73	Rapidly progressing Cushing syndrome - Virilisation (clitoromegali + severe hirsutism) - Hipertension 220/120 mmHg	Increased Ürinary and plasma cortisol- Urinary Free cortisol-Testosterone - Estradiol − 17-OHP Low: FSH ve LH	Low < 10 pg / mL	No Adrenal Mass No ovarian mass Venous catheter shows left ovarian origin	Left ovarian mass Atrophic right ovary	2 × 2 × 1 cm lipit hücreli tümör, ancak kesin malignite kanıtı yok	No adjuvant treatment - Recurrence after 12 months peritoneal and omental metastases
Farida vd(2016) (17)	34	Cushing syndrome - Psychiatric problems - Hirsutism - Hypertension - Diabetes mellitus - Lower extremity edema - Pelvic Mass	High: - Cortisol - Estradiol - Testosterone − 17-OHP	Low < 10 pg / mL	No Adrenal Mass Small adrenals Ovarian tumor	- Ovarian tumor: -Hemoperitoneum - Lymph nodes	- Ovarian tumor 14 × 13 cm- Peritoneum and lymph node metastases	No adjuvant treatment, death post op day two due to pulmonary embolism
2019 Case	21	Cushing syndrome - Psychiatric problems - Hirsutism – paraplegia- diabetes mellitus - pelvic mass	High: - Plasma and free urine cortisol-	HighACTH (89 pg/m l)	Bilateral ovarian mass, No Adrenal Mass Normal adrenals	bilateral adneksıyel kitle batın içi ek patoloji yok	11x6x5 cm and 9x8x5 cm sized SCLT of ovaries left and right respectively	Normalization of steroids - Metastasis to the brain on month 3, Radiotherapy and chemotherapy ongoing

Since SLCT can cause metabolic disorders such as severe hypokalemia and ketoacidosis, the prognosis may be poor. However, the most important causes of mortality are metastatic disease and thromboembolic events.(Van der Pas et al., 2013) Five cases reported in the literature resulted in exitus of the patient. (Marieb et al., 1983, Young and Scully, 1987, Donovan et al., 1993, Elhadd et al., 1996) In our case, there was a serious risk of thromboembolism due to severe hypokalemia and immobility. Clinical improvement was observed only after the operation despite aggressive replacement therapy. Anticoagulant agents and pneumatic compression devices were used for thromboembolism prophylaxis.

## 4 Conclusıon

In conclusion, SCLT with Heterologous Elements is a rare histological subtype of ovarian SCLTs manifested by hormone-related symptoms or non-hormonal symptoms. As in our case, ACTH-independent Cushing syndrome can be seen as an ectopic tumor that produces glucocorticoids and sex hormones. The treatment is surgical resection and fertility-sparing conservative surgeries may be preferred for young patients with stage 1 disease. However, the picture due to endocrine causes is often malignant and complete resection is required.

In the patient we presented in our case, ovarian tumors detected in abdominal CT scans were confirmed by histological and immunohistochemical staining and normalization of metabolic and endocrine activity immediately after ovarian tumor resection showed that ovarian tumors may cause CS.

## Author contributions

Gynecological surgeon Dr. Tuba OFLI, gynecological oncologist Dr. He performed the surgery with Gurkan KIRAN. Dr Atilla KUNT provided 3rd assistance to the surgery. Dr. Tuba wrote the draft. Dr. Tuba, Dr. Gurkan and Dr. Arilla edited the draft. Dr. Gurkan approved the draft. All authors have read, revised and approved the article.

## Declaration of Competing Interest

The authors declare that they have no known competing financial interests or personal relationships that could have appeared to influence the work reported in this paper.

## References

[b0005] Bhansali A., Walia R., Rana S.S., Dutta P., Radotra B.D., Khandelwal N., Bhadada S.K. (2009). Ectopic Cushing syndrome: experience in a tertiary care center. Indian J. Med. Res..

[b0010] Terzolo, M., Bovio, S., Pia, A., Reimondo, G., Angeli, A. 2009. Treatment of adrenal incidentaloma. En Terzolo Best Practice and Research: Clinical Endocrinology and Metabolism. 10.1016/j.beem.2009.04.001. 10.1016/j.beem.2009.04.001 19500766

[b0015] Colombo N., Parma G., Zanagnolo V., Insinga A. (2007). Gabriella parma management of ovarian stromal cell tumors. J. Clin. Oncol..

[b0020] Colombo N., Parma G., Zanagnolo V., Insinga A. (2007). Management of ovarian stromal cell tumors. JCO.

[b0025] Demidov, V.N., Lipatenkova, J., Vikhareva, O., Van Holsbeke, C., Timmerman, D., Valentin, L. 2008. Imaging of gynecological diseases (2): Clinical and ultrasound features of Sertoli cell tumors, Sertoli-Leydig cell tumors and Leydig cell tumors. Ultrasound Obstet Gynecoli 2008; 31: 85 - 91. Wiley Online LibraryCASPubMedWeb of Science®Google Scholar. 10.1002/uog.5227 18098335

[b0030] Donovan J.T., Otis C.N., Powell J.L., Cathcart H.K. (1993). Cushing's syndrome secondary to malignant lipoid cell tumor of the ovary. Gynecol. Oncol..

[b0035] Elhadd T.A., Connolly V., Cruickshank D., Kelly W.F. (1996). An ovarian lipid cell tumor that causes virilization and Cushing's syndrome. Clin. Endocrinol..

[b0040] Louiset, E., Gobet, F., Libe, R., Horvath, A., Renouf, S., Cariou, J., Rothenbuhler, A., Bertherat, J., Clauser, E., Grise, P., Stratakis, C.A. 2010. micronodular adrenal hyperplasia and ectopic adrenocortical adenoma ACTH independent Cushing syndrome. J. Clin. Endocrinol. Metabol., 2010, 95, 18-24. (doi: 10.1210 / jc.2009-0881). 10.1210/jc.2009-0881 PMC2805485 19915020

[b0045] Marieb N.J., Spangler S., Kashgarian M., Heimann a Schwartz M.L., Schwartz P.E. (1983). Cushing's syndrome secondary to ectopic cortisol production with ovarian carcinoma. J. Clin. Endocrinol. Metab..

[b0050] Marieb N.J., Spangler S., Kashgarian M., Heimann A., Schwartz M.L., Schwartz P.E. (1983). Cushing’s syndrome secondary to ectopic cortisol production by an ovarian carcinoma. J. Clin. Endocrinol. Metab..

[b0055] Pollack R.P., Brett E. (2009). ACTH independent Cushing syndrome that occurs during pregnancy. Endocrine Administration..

[b0060] Stacher E., Pristauz G., Scholz H.S., Moinfar F. (2010). Sertoli-Leydig cell tumors with heterologous elements associated with unilateral serous cystadenoma - Case report. Int J Gynecol Pathol.

[b0065] Tavassoli, F.A., Mooney, E., Gersell, D.J., et al. 2003. Sertoli-leydig cell tumors. In: Tavassoli, F.A., Devilee, P, (Eds)., World health organization classification of IARC Press Tumors, Pathology and Genetics. Lyon: Tumors of breast and female genital organs; [Google Scholar].

[b0070] Van der Pas R., Leebeek F.W., Hofland L.J., de Herder W.W., Feelders R.A. (2013). Hypercoagulability in Cushing's syndrome: prevalence, pathogenesis, and treatment. Clin. Endocrinol..

[b0075] Young R.H., Scully R.E. (1987). Ovarian steroid cell tumors associated with Cushing's syndrome: a report of three cases. Gynecol. Pathol. Int. J..

